# Monocyte as a prognostic marker in patients with idiopathic pulmonary fibrosis

**DOI:** 10.1186/s12931-021-01869-8

**Published:** 2021-10-21

**Authors:** Shiping Zhu

**Affiliations:** Department of Respiratory Medicine, Hangzhou Hospital of Traditional Chinese Medicine, No. 453, Tiyuchang Road, Hangzhou, Postal-code: 310000 Zhejiang China

**Keywords:** Monocyte, Idiopathic pulmonary fibrosis, Regression model, Mortality

## Abstract

This letter raised some concerns about the study by Karampitsakos et al. in a recent issue of Respiratory Research.

## To the Editor,

We read the interesting study [1] by Karampitsakos et al., which investigated the predictive value of monocyte and red cell distribution width in patients with idiopathic pulmonary fibrosis. In the multivariable regression model, the monocyte and red cell distribution width were converted into dichotomous variables according to their respective cut-off values (0.6 k/ul and 14.1). However, after adjusting for confounders, both monocyte and red cell distribution width were not significantly associated with FVC%pred. We have several concerns about the linear regression model.

First, confounding bias is crucially important in observational studies. However, in the current regression model, the selection of confounding factors seems confusing, as almost all the included factors were non-significant except for gender. Second, we noted that the smoker status including current smoker (n = 38), ever smoker (n = 199) and never smoker (n = 63) are mutually exclusive (38 + 199 + 63 = 300). Statistically, these three variables should be included in the regression model as a dummy variable, meaning that one of them is supposed to be the reference level (coefficient = 0). However, in the current model, the coefficients for the current smoker, ever smoker, and never smoker were 19.3, 9.4, and 6.3, respectively. This needs to be verified. Third, a total of 14 variables were included in the model for FVC%pred. However, even some known risk factors (such as age) [2, 3] are also non-significant in the model. Therefore, the risk of multicollinearity should be evaluated [4, 5], as the presence of multicollinearity can lead to serious statistical problems in parameter estimation, such as non-significant coefficients, high standard errors, or ‘‘wrong’’ sign. Forth, in the Kaplan–Meier survival curve, high monocyte was significantly associated with increased mortality in the derivation cohort while became non-significant in the validation cohort. This result may be affected by the proportion of patients with extremely high monocyte count (> 0.95 k/ul). In another similar study [6] with large sample size (n = 2067), all patients were divided into three groups based on monocyte count (< 0.6, 0.6–0.95, > 0.95 k/ul) and the proportion of patients with extreme high monocyte (> 0.95 k/ul) is very small (50/2067 = 2.4%). In the Kaplan–Meier for all-cause mortality (Fig. 1C of Kreuter et al.’s study), the mortality was significantly higher in the group with extremely high monocyte (> 0.95 k/ul), while it was close in groups with monocyte < 0.6 k/ul and 0.6–0.95 k/ul. In the validation cohort of the current study, the sample size is small (n = 189). Therefore, it is reasonable to conclude that the non-significant mortality rate between low and high monocyte (< 0.6 vs. > 0.6 k/ul) may be caused by the close mortality rate and low proportion of extremely high monocyte count.

## Data Availability

Not applicable.

## Abbreviation

FVC: Forced vital capacity
